# Case Report: A case of Crohn's disease with right atrial thrombosis

**DOI:** 10.3389/fped.2024.1353883

**Published:** 2024-03-21

**Authors:** Zehang Hu, Yi Gao, Shumin Fan

**Affiliations:** Department of Ultrasound, Shenzhen Children's Hospital of China Medical University, Shenzhen, China

**Keywords:** Crohn’s disease, right atrial thrombosis, echocardiography, contrast-enhanced ultrasound, children

## Abstract

Crohn's disease (CD) is a chronic, non-specific inflammatory disease of the intestinal tract with an unknown etiology. It presents with clinical symptoms such as abdominal distension, abdominal pain, diarrhea, bloody stools containing mucus or pus, and other manifestations. CD has a prolonged and chronic course and can lead to various complications that significantly impact patients’ quality of life. Patients with CD have hypercoagulable blood and are prone to thromboembolic diseases, which pose a serious threat to their lives. Several studies have indicated that inflammatory bowel disease is a risk factor for venous thromboembolism. The pathogenesis involves abnormalities in the coagulation-anticoagulation system, fibrinolytic system, platelets, vascular endothelial dysfunction, as well as the effects of therapeutic agents. In this case report, we present a rare case of a 15-year-old female patient with active CD complicated by the presence of a right atrial thrombus. Laboratory tests revealed abnormalities in both the coagulation-anticoagulation system and fibrinolysis system in the patient. The initial diagnosis, based on transthoracic echocardiography and contrast-enhanced echocardiography, confirmed the presence of a thrombus in the right atrium. Subsequent administration of anticoagulant and thrombolytic therapy resulted in gradual reduction in size until complete disappearance, as evidenced by dynamic monitoring. Ultrasound examination is considered as the preferred method for follow-up evaluation in patients with CD due to its ability not only to assess gastrointestinal complications but also to aid early identification of cardiovascular complications, thereby enabling timely intervention and treatment—which remains our primary focus of research and effort.

## Introduction

Inflammatory bowel disease (IBD) is a chronic, non-specific inflammatory disorder of the gastrointestinal tract, encompassing Crohn's disease (CD) and ulcerative colitis (UC). CD is the predominant form of chronic IBD in children, characterized by segmental granulomatous inflammation that affects the entire intestinal wall. It is commonly localized in the terminal ileum and ascending colon. The pathogenesis of CD may be associated with genetic susceptibility, dysbiosis of gut microbiota, impaired intestinal mucosal barrier function, dysregulation of innate and adaptive immune responses, as well as external environmental factors (1). Clinical manifestations of CD include gastrointestinal and systemic symptoms. Among the complications related to CD, venous thromboembolism (VTE) is a relatively uncommon but significantly life-threatening condition that can increase mortality rates. Population-based studies from Canada, Europe, and Taiwan have demonstrated an elevated risk of VTE in IBD patients compared to healthy individuals. This risk closely correlates with disease activity levels (2–5). Currently, to the best of our knowledge, there have been limited reported cases of concurrent right atrial thrombus in pediatric patients with active CD. In this study, we present a case of a child with active CD who developed a right atrial thrombus, initially diagnosed by echocardiography. The thrombus gradually decreased in size and eventually resolved following anticoagulation and thrombolysis treatment, thereby confirming the diagnosis made by echocardiography. We anticipate that the findings from this case report will contribute to early clinical diagnosis and intervention for similar cases in the future. This study is reported in accordance with the CARE guidelines (6).

## Case report

A 15-year-old female, presented with recurrent oral ulcers for over 3 years and was admitted for scheduled treatment. The patient developed idiopathic oral ulcers more than 3 years ago, accompanied by vulvar and perianal ulcers suggestive of Behçet's disease. Treatment was initiated but the ulcers persisted intermittently. One year ago, the patient experienced intermittent left upper abdominal pain along with worsening oral and perianal ulcers. She was hospitalized at our institution more than nine months ago for further evaluation including small bowel endoscopy, diagnosed CD, with lesions located in the end of the ileum and the colon. Subsequently, she underwent six infliximab treatments; however, she discontinued medication on her own accord and subsequently experienced mild abdominal pain accompanied by anal discomfort. After resuming medication administration again, her symptoms improved. She is currently admitted for follow-up colonoscopy examination. And she had a history of non-compaction of ventricular myocardium. Since onset of symptoms, the patient has been experiencing bowel movements once every one to two days with yellowish loose stools; otherwise no specific abnormalities were noted in stool characteristics.

Physical examination revealed no cardiac heave or lift in the precordium; apex beat located at the left fifth intercostal space midclavicular line without any lifting sensation or palpable thrill; relative cardiac dullness within normal limits; heart rate of 70 beats per minute with strong regular rhythmical heart sounds; no murmurs heard upon auscultation across various valve areas nor any signs of pericardial friction rub.

Specialized examination showed flat abdomen without visible gastric or intestinal contour deformities or abdominal wall varices; soft abdominal wall without tenderness upon palpation in lower abdomen nor rebound tenderness observed; no masses detected on palpation.

Laboratory tests are shown in Table 1.

**Table 1:** Laboratory test results of the patient under continuous monitoring.

	hs-CRP (mg/L) (1–10)	PLT (/L) (188–472 × 10^9)	WBC (/L) (4.1–11.0 × 10^9)	Hb (g/L) (114–154)	APTT (S) (32.2–49.1)	PT (S) (10.5–14.5)	TT (S) (13.2–20.1)	Fibrinogen (g/L) (1.31–4.51)	D-dimer (ug/ml) (0.000–0.500)
First day after hospitalization	3.50	201	3.78	122	38.4	14.8	16.0	3.7	/
4th day after hospitalization	/	194	3.46	118	51.9	16.5	70.8	2.62	1.64
5th day after hospitalization	/	186	3.88	113	71.6	17.0	238.0	1.63	/
6th day after hospitalization	/	160	3.32	115	149	17.4	>240.0	0.99	2.74
7th day after hospitalization	/	174	3.34	115	51.7	27.7	38.1	<0.20	/

PLT, refers to platelets; WBC, stands for white blood cells; Hb, represents hemoglobin; APTT, denotes activated partial thromboplastin time; PT, represents plasma prothrombin time; TT, stands for thrombin time. hs-CRP, represents hypersensitive C-reactive protein.

A comparative enema was performed on the first day of admission to assess the presence of stenosis in the lower digestive tract and the thickening of the intestinal wall. On the large intestine shows no evidence of local luminal narrowing following rectal infusion with saline solution. The wall of the ascending colon, ileocecal region, and terminal ileum demonstrates irregular thickening characterized by distinct layering. Notably, there is enhanced echogenicity in the submucosal layer which measures up to 0.6 cm in thickness. Thickening can be observed in both sides' soft tissues surrounding the anus; although there is decreased echogenicity present it remains relatively homogeneous in appearance. Furthermore, color Doppler flow imaging reveals punctate blood flow signals within these thickened intestinal walls.

**Figure 1:**
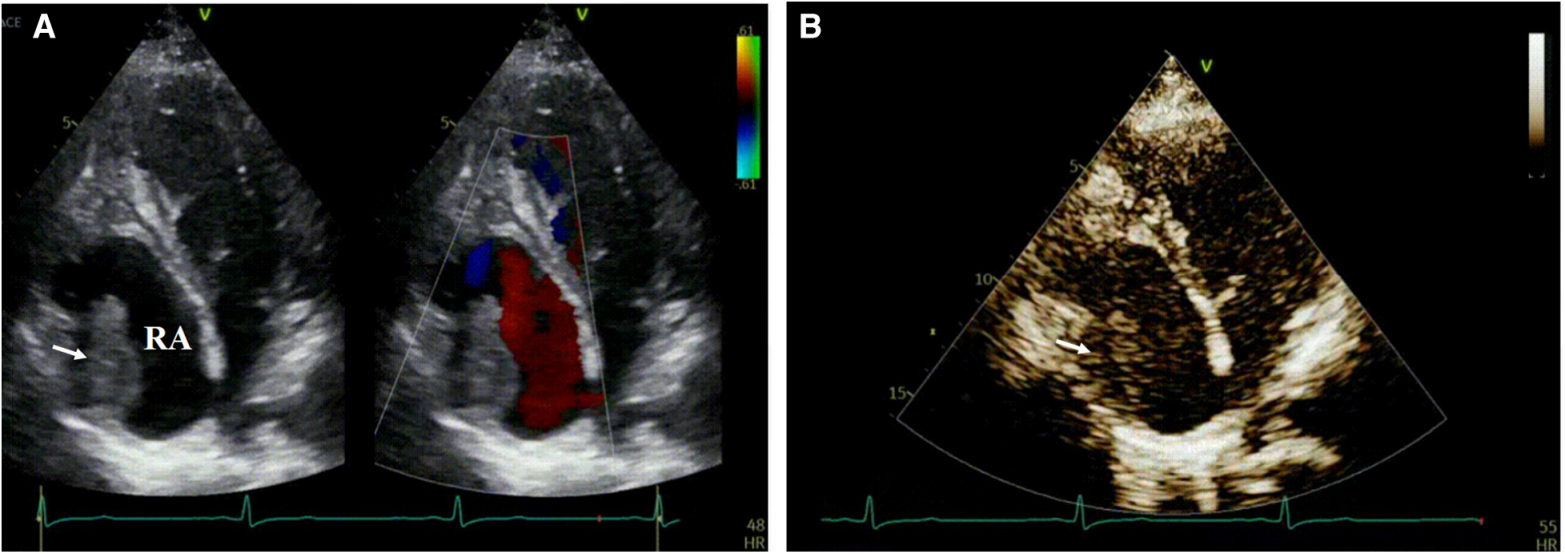
Depicts the right atrium in transthoracic echocardiography with the annotation that RA represents the right atrium. In (**A**) an echogenic mass is visualized within the right atrium (indicated by arrows), while color Doppler flow imaging reveals no blood flow signal within the mass. (**B**) Demonstrates that contrast-enhanced cardiac imaging does not exhibit significant enhancement of the aforementioned mass.

Since the patient had a history of non-compaction of ventricular myocardium, transthoracic echocardiography was performed to assess cardiac function. On the third day of admission, TTE revealed an irregularly shaped mass with low echogenicity measuring approximately 43 mm × 26 mm and attached to the right atrial wall. The mass had a broad base measuring about 35 mm and was adjacent to the tricuspid annulus (Figure 1A). Following intravenous administration of contrast agent (Sonovue), no significant enhancement was observed within the mass. Based on ultrasound findings, it is speculated that there is a thrombus in the right atrial appendage (Figure 1B).

**Figure 2:**
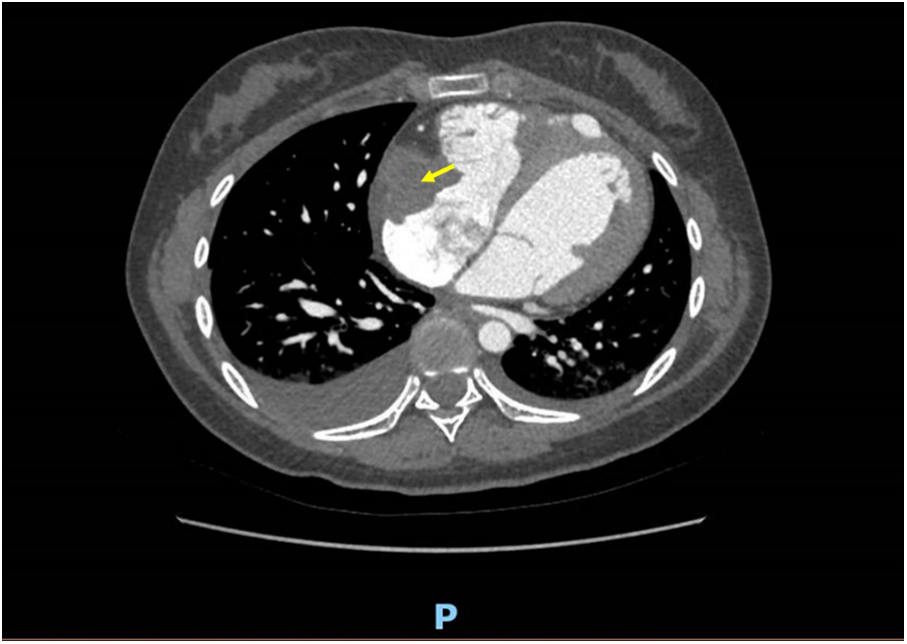
A well-defined, mass-like soft tissue density lesion measuring approximately 30 mm × 34 mm × 32 mm (anteroposterior diameter × transverse diameter × cranio-caudal diameter) is observed at the right atrial appendage and tricuspid valve chordae tendineae. No discernible enhancement is noted on contrast-enhanced imaging.

On the fourth day of hospitalization, an enhanced cardiac CT scan revealed a well-defined oblong soft tissue density shadow measuring approximately 30 mm × 34 mm × 32 mm (anteroposterior diameter × transverse diameter × cranio-caudal diameter) within the right atrium and its appendage. The density of the shadow was around 30 Hounsfield units, and no significant enhancement was observed after contrast agent injection, raising suspicion for thrombus formation (Figure 2). Dynamic electrocardiogram reveals sinus bradycardia, an accelerated ventricular escape rhythm, occasional atrial and ventricular premature beats, ST changes, and no significant abnormalities in heart rate variability.

After admission, both cardiac ultrasound and CT enhancement findings suggest the possible presence of a right atrial thrombus, indicating a severe condition. Immediate initiation of anticoagulant therapy is warranted with intravenous infusion of heparin at a rate of 18U/kg/h. Following transfer to the cardiovascular department, the patient's condition deteriorates significantly, necessitating maintenance treatment with intravenous injection of sodium heparin at a dose of 100U/Kg/h, in combination with urokinase for thrombolysis. Simultaneously, oral administration of warfarin as an anticoagulant is initiated to dynamically monitor the child's coagulation function and hematological changes during thrombolysis, while regular follow-up cardiac ultrasounds are scheduled to monitor clot changes. A repeat echocardiogram performed after 7 days reveals gradual reduction in clot size, which completely resolves after 20 days, allowing for discharge under observation.

## Discussion

CD is the most prevalent chronic IBD in children, typically occurring after the age of 10. It is characterized by segmental granulomatous inflammation that affects the entire thickness of the intestinal wall and can involve any part of the digestive tract, although it primarily affects the terminal ileum and ascending colon. The etiology remains unknown, and clinical manifestations include gastrointestinal and systemic symptoms. Gastrointestinal complications may include intestinal obstruction, fistulas, anal fistulas, intra-abdominal abscesses or masses. Moreover, approximately 25% of patients exhibit various extraintestinal manifestations such as joint involvement, mucocutaneous lesions, oral ulcers, eye involvement, and thrombosis formation. In 2008, the American College of Chest Physicians clearly stated that IBD is a risk factor for VTE (7). Patients with CD have a threefold higher risk of developing blood clots compared to healthy individuals. During active periods of disease activity, this risk increases up to sixteen times higher than normal levels. Autopsy reports indicate that thromboembolic events occur at rates as high as 39%−41%, with pulmonary embolism and deep vein thrombosis accounting for 90.4% of cases; however other vessels such as mesenteric veins, portal veins retinal veins and some arteries can also be affected. Studies showed that pediatric and adolescent IBD patients have an increased risk of VTE (8, 9) and female pediatric IBD patients were found to be at a high risk of developing VTE (10).

In this case, the patient is a female child with CD and is therefore at risk for thrombosis. Independent risk factors for increased risk of VTE in IBD include: long-distance journey, postoperative status, injury/immobility, pregnancy/delivery, oral contraceptives/hormone substitution, etc (11). CD patients exhibit a thrombosis formation mechanism characterized by the following factors (12): ① Abnormal coagulation-anticoagulation system, with increased levels of procoagulant substances and decreased levels of anticoagulant substances in plasma (12, 13); ② Aberrant fibrinolysis system, as evidenced by elevated levels of D-dimer and fibrin degradation products during active periods, indicating recent or ongoing intravascular coagulation (14); ③ Platelet abnormalities, including increased platelet count and reduced volume in circulating blood during active periods. Inflammatory substances from both the bloodstream and local intestinal inflammation act on platelets as mediators in the clotting process (15, 16); ④ Endothelial dysfunction caused by intestinal inflammation leads to heightened expression levels of vascular endothelial growth factor and other factors. This disrupts endothelial barrier function, directly damages intestinal microbiota epithelial cells, and increases adhesion between endothelial cells, leukocytes, and platelets (17); ⑤ Influence of therapeutic drugs: The use of corticosteroids such as glucocorticoids poses a potential risk factor for postoperative VTE formation in patients.

The child in this case had a medical history of CD and was in the active phase of CD (hypersensitive C-reactive protein > 3 mg/L) and the pediatric CD activity index (PCDAI) is 10. CD remission is best defined by a score <10 points or <7.5 points (18, 19). Therefore, the patient has risk factors for thrombosis, and it is necessary to perform thromboembolic screening. And laboratory tests revealed elevated levels of D-dimer, activated partial thromboplastin time, prothrombin time, and thrombin time in the plasma. These findings indicate abnormalities in the coagulation-anticoagulation system and fibrinolysis system that resulted in the formation of an intracardiac thrombus. Initially diagnosed by TTE as a right atrial thrombus, contrast-enhanced ultrasound showed no enhancement within the mass, suggesting thrombosis. Dynamic observation demonstrated gradual reduction of the mass until its complete disappearance with anticoagulant therapy. It is important to differentiate intracardiac thrombi from myxomas and other lesions such as vegetations. In addition to two-dimensional imaging and contrast-enhanced echocardiography examination, dynamic observation also proves beneficial. Anticoagulant treatment leads to progressive shrinkage or resolution of the thrombus; however, vegetations and myxomas do not exhibit significant changes on sonograms in the short term.

## Conclusion

In conclusion, patients with CD exhibit a prothrombotic state in their blood, thereby elevating the risk of thromboembolic diseases and potentially exacerbating the condition. Hence, it is imperative to consider this aspect during the diagnosis and treatment of CD. Ultrasound examination serves as the preferred modality for follow-up in CD patients due to its ability not only to assess intestinal lesions and gastrointestinal complications such as strictures, obstructions, fistulas, perianal abscesses, cellulitis, and anal fistulas but also to evaluate cardiovascular complications including thrombosis.

## Data Availability

The original contributions presented in the study are included in the article/Supplementary Material, further inquiries can be directed to the corresponding author.
